# A simple and inexpensive laser dissection of fasciculated axons from motor nerve organoids

**DOI:** 10.3389/fbioe.2024.1259138

**Published:** 2024-01-29

**Authors:** Yasuhiro Ikegami, Tomoya Duenki, Ikuma Arakaki, Ryo Sakai, Tatsuya Osaki, Satoshi Ashihara, Tsuyoshi Furushima, Yoshiho Ikeuchi

**Affiliations:** ^1^ Institute of Industrial Science, The University of Tokyo, Tokyo, Japan; ^2^ Institute for AI and Beyond, The University of Tokyo, Tokyo, Japan; ^3^ Department of Chemistry and Biotechnology, Graduate School of Engineering, The University of Tokyo, Tokyo, Japan; ^4^ Laboratory for Integrated Micro Mechatronic Systems, National Center for Scientific Research-Institute of Industrial Science (LIMMS/CNRS-IIS), The University of Tokyo, Tokyo, Japan

**Keywords:** organoids, laser, tissue engineering, PDMS, axons

## Abstract

Motor nerve organoids could be generated by culturing a spheroid of motor neurons differentiated from human induced pluripotent stem (iPS) cells within a polydimethylsiloxane (PDMS) chip which guides direction and fasciculation of axons extended from the spheroid. To isolate axon bundles from motor nerve organoids, we developed a rapid laser dissection method based on localized photothermal combustion. By illuminating a blue laser on a black mark on the culture device using a dry-erase marker, we induced highly localized heating near the axon bundles. Moving the laser enabled spatial control over the local heating and severing of axon bundles. This laser dissection requires a black mark, as other colors did not produce the same localized heating effect. A CO_2_ laser destroyed the tissue and the device and could not be used. With this simple, economical laser dissection technique, we could rapidly collect abundant pure axon samples from motor nerve organoids for biochemical analysis. Extracted axonal proteins and RNA were indistinguishable from manual dissection. This method facilitates efficient axon isolation for further analyses.

## 1 Introduction

During development of the nervous system, neurons spontaneously form functional circuits by extending axons which are thin and long protrusions specialized in transmitting action potentials toward their target cells. The shaft of an axon could be as thin as sub-micron in diameter and have limited cytoplasm, specialized in efficient transduction of long-distance signals within the body. Presynaptic sites of axons are enriched with apparatus for releasing neurotransmitters, enabling synaptic transmissions. A majority of these molecular and sub-cellular components of axons are actively trafficked from the cell body. Additionally, local synthesis and degradation of proteins within axons contributes to acute and localized response and maintenance of axonal proteome (Jung et al., 2012; Kim and Jung, 2020), although dynamics of axonal proteostasis is yet to be determined. Degradation of neurons in early stages of neurodegenerative diseases including amyotrophic lateral sclerosis (ALS) are suspected to start within axonal components but the biochemical mechanisms of axonal dysregulations in neurodegenerative diseases are not fully understood yet (LaMonte et al., 2002; Morfini et al., 2009; Bilsland et al., 2010; Alami et al., 2014; Ito et al., 2016). Therefore, biochemical analysis of axons is essential for understanding physiology and dysregulation of axons, however, obtaining highly purified axons in large quantities is technically challenging. For instance, nerves and axon tracts dissected from *in vivo* tissues contain a substantial amount of non-neuronal cells which interfere with isolation of axons.

To obtain axons from neurons cultivated *in vitro*, various methods have been invented. Since axons are thinner and longer than the cell bodies and dendrites, axons can be isolated by their size. Axons can be isolated using a membrane with small pores or a micro device with thin slits (Taylor et al., 2005; Taylor et al., 2010; Unsain et al., 2014; Fantuzzo et al., 2019; Luo et al., 2021; Spijkers et al., 2021). Pores and slits are designed so that only axons but not cell body and dendrites can pass through, which then can be collected for analysis. Low amount of RNA could be obtained with the collected axons (Nijssen et al., 2019). Axons can also be isolated from three-dimensional neuronal tissues (a.k.a. “Neuron balls”) extending axons from aggregated cell bodies (Parvin et al., 2019; Sharma et al., 2019). Neurons are aggregated into a ball-like structure and then plated on a surface where neurons can extend axons radially. By physically removing the neural aggregates, the axons attached and left on the culture surface can be obtained. These techniques are well-established and widely used, however, they are difficult to scale up to consistently obtain large quantities of axons with reasonable labor and time.

Axons assemble and form a bundle in many parts of the nervous system, including peripheral nerves and corpus callosum (Stewart, 2003; Ahsan et al., 2007). Axo-axonal interaction facilitates fasciculation of axons, which provides them with a more physiological microenvironment compared to dispersed cultures (Pollerberg et al., 2013; Siegenthaler et al., 2015; Kirihara et al., 2019). To mimic the fasciculated axons *in vitro*, we have developed a technique to construct motor nerve-like tissues bearing a unidirectional axon bundle using a microfluidic device. This device is equipped with a chamber that receives aggregated neurons and a channel in which axons extend from the neurons (Kawada et al., 2017; Kirihara et al., 2019; Osaki et al., 2020). After placing the neurons into the device, axons first grow within the chamber, then they grow into the channel due to physical restriction of their growth in the device. The axons in the channel spontaneously assemble into a bundle within which axons align unidirectionally. Since the bundle structure of axons mimics the motor nerves bridging the spinal cord and the skeletal muscles, we named the tissues motor nerve organoids (MNOs). The cell bodies and dendrites stay aggregated in the three-dimensional tissue while axons grow away from them. The axon bundle is long (can be over a centimeter) and thick (over 100 μm in diameter), which makes it a good source of abundant axons for biochemical analyses.

To isolate the axon bundles from cell bodies, bundles have been cut manually using forceps or micro scissors in the past (Guertin et al., 2005; Kawada et al., 2017). However, the procedure requires skills and training, which could provide inconsistency and variability. Furthermore, manual processing requires substantial time which could negatively impact the quality of axons after they are isolated. To develop an easy and fast alternative method, we turned our attention to laser cutting techniques. Laser cutting technique contributes significantly to precision processing in a variety of industrial fields, owing to its accuracy and speed (Ueda et al., 1990). Carbon dioxide (CO_2_) gas laser is highly versatile and has been widely used for processing various materials including transparent glasses, plastics, rubbers (Caiazzo et al., 2005). Affordable CO_2_ laser systems are sold for cutting and engraving materials in a few thousands U.S. dollars. At the same time, blue lasers are gaining popularity in material processing and are even less expensive than CO_2_ laser systems that are commercially available. Compared to the 10.6 μm wavelength of CO_2_ lasers, blue lasers use substantially shorter wavelength of around 400 nm. This allows the blue light to be tightly focused and to cut target materials more accurately without substantial heat and damage. Moreover, a blue light laser has higher selectivity of target materials based on absorption spectrum, which could give it specificity but also restrict its applications.

In this study, we developed a simple and inexpensive method for cutting axonal bundles using a blue light laser to improve reproducibility, accuracy and speed of isolation of axons.

## 2 Experimental section

### 2.1 Motor nerve organoids cultured in a PDMS culture chips

**FIGURE 1:**
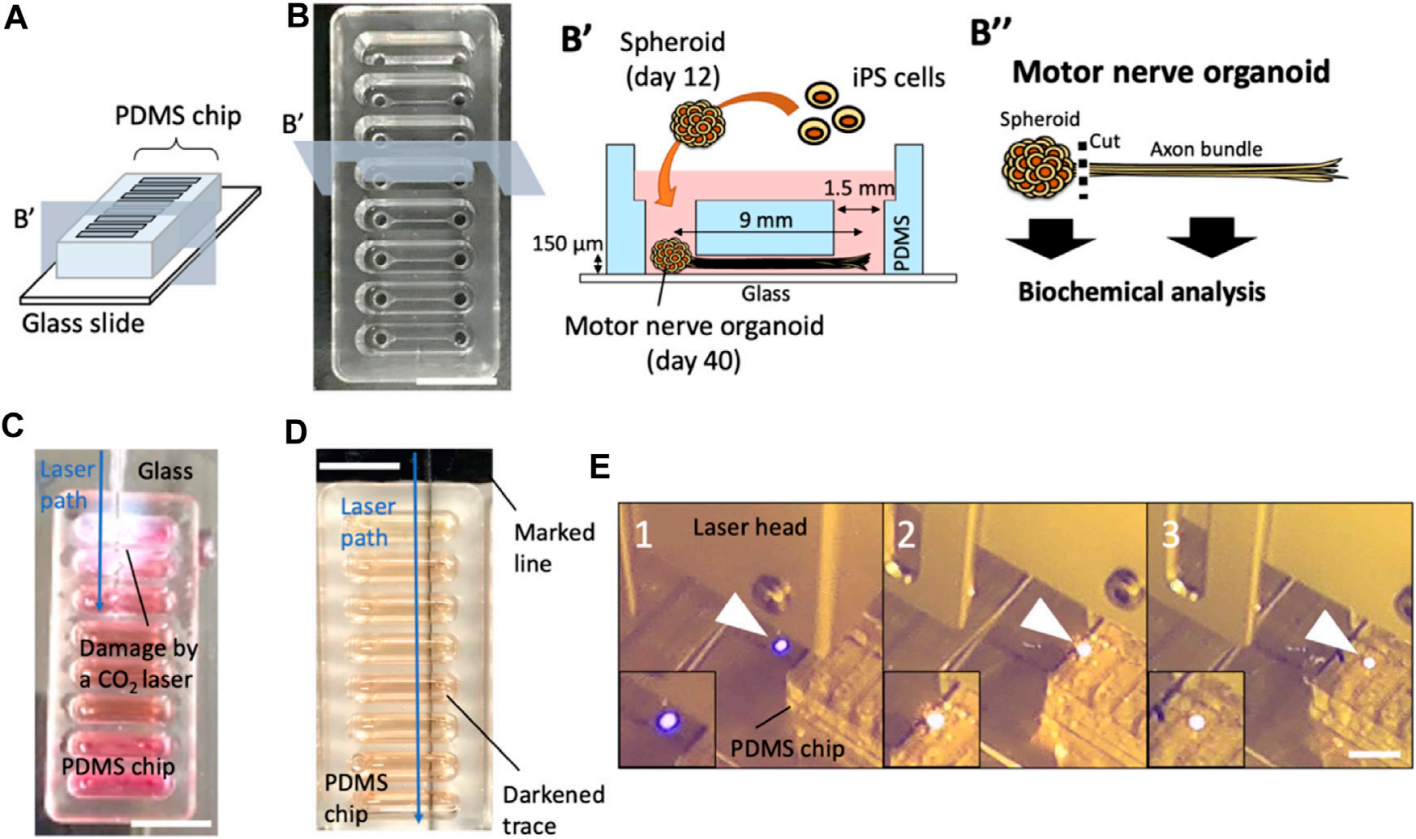
Effects of laser cutters on PDMS culture devices. **(A)** Schematic of a PDMS chip. **(B)** A PDMS culture chip and a schematic of PDMS chip cross section and experimental procedure of motor nerve organoid generation. **(C)** A CO_2_ laser-induced damage on a culture device. **(D)** A marked line next to the PDMS chip induced a dark trace upon blue laser exposure. **(E)** Laser-exposed spot on a colorless surface (e.g., glass). is normally blue (1). When the laser hits the black marking, the exposed spot turns orange (2). As the laser moves, it continues to generate the orange spot at the exposure site (3), moving the orange spot (laser exposed local combustion site) into the PDMS device. Arrowheads indicate laser-exposure sites. Scale bars: 1 cm.

Polydimethylsiloxane (PDMS) culture chips were fabricated at Fukoku Bussan, Japan by a standard transfer molding process (Figures 1A, B). The PDMS chip has 8 units, with each unit containing 2 holes that are connected by a 150 µm thick microchannel. Center-to-center distance between the two holes is 9 mm and inner diameter of each hole is 1.5 mm. The PDMS chip was placed on a glass slide (S2112, Matsunami Glass Ind., Ltd., Osaka, Japan) for culturing motor nerve organoids (MNOs).

30HU-002 human induced pluripotent stem (iPS) cells were purchased from iXCells (California, United States). The iPS cells were maintained on a Matrigel-coated dish with mTeSR Plus (#05825, STEMCELL Technologies, British Columbia, Canada). MNOs were generated using the previously described protocol (Osaki et al., 2020). Briefly, iPS cells dissociated with TrypLE were seeded into a low-adhesion 96-well U bottom plate at 40,000 cells/well in 100 µL of mTeSR Plus supplemented with 10 µM Y-27632 (Rock inhibitor, FUJIFILM Wako Pure Chemical Corporation, Osaka, Japan). The differentiation was initiated in DMEM/F12 medium (Sigma-Aldrich, St. Louis, United States) supplemented with 15% Knockout Serum Replacement (Thermo Fisher Scientific, Massachusetts, United States), 1% GlutaMAX (Gibco), 1% non-essential amino acids (NEAA, Sigma), 10 µM SB431542 (Wako), and 100 nM LDN-193189 (Wako) for the initial 2 days of culture. The medium was gradually switched to N_2_ medium (Neurobasal medium supplemented with 1% N_2_ supplement, 1% GlutaMAX, and 1% NEAA) for the following 8 days of culture. During this period, cells were treated with 1 μM retinoic acid (RA), 1 μM Smoothened agonist (SAG, Wako), 5 μM SU-5402 (Sigma), and 5 μM DAPT (Sigma). On day 12, the resultant motor neuron spheroid was transferred into the microfluidic device with maturation medium (Neurobasal medium supplemented with 2% B27 supplement (with vitamin A), 1% GlutaMAX, and 1% Penicillin/Streptomycin) containing 20 ng/mL BDNF.

Prior to the tissue culture procedure, the PDMS chips were placed on glass slides, disinfected with 70% ethanol and dried. The internal surface of microchannel in PDMS chip and glass slide were coated with Matrigel in DMEM/F12 (1:50) for 1 h at room temperature. The coating solution was replaced with 150 uL of maturation medium, then the spheroid was placed into the culture device using a wide-bore tip. PDMS chip was placed inside a OmniTray Single-Well plate (Thermo Scientific Thermo Fisher Scientific, Massachusetts, United States) and cultured in an incubator (5% CO_2_, 37 °C) for up to 4 weeks (a total culture period of 40 days). During this period, axons extend from the spheroid into the microchannel and spontaneously assemble into an axon bundle, which results in the formation of a motor nerve organoid (MNO). Half of the culture medium was replaced with fresh maturation medium every 2–3 days.

### 2.2 Laser cutting of the motor nerve organoids

We employed a CO_2_ laser cutter (laser power: 30 W, laser diameter: 0.2 mm, laser intensity: 95 kW/cm^2^, Etcher Laser Pro, smartDIYs Co., Ltd. Yamanashi, Japan) and a blue laser cutter (laser power: 3.5 W, laser diameter: 0.2 mm, laser intensity: 11 kW/cm^2^, Etcher Laser, smartDIYs). We inverted the blue laser cutter and drilled a hole to place a microscope slide holder (universal mounting frame M, Zeiss, Germany) (Figures 3A–C). The slide holder was covered to eliminate light leakage. A small segment of the bottom surface of the culture devices was painted with a marker (Dry Erase Marker, #B07RYTSNDV, Shuttle Art, Hangzhou, China). The absorbance spectra of the markers were measured with UV-VIS-NIR absorption spectrometer (V-670, JASCO Corporation, Tokyo, Japan). The PDMS device was placed into slide holder and exposed to the blue light laser (λ: 445 nm, cutting speed: 100 mm/min). To check local temperature of the irradiated samples, we used an IR camera (Xi 400, Optris GmbH, Berlin, Germany).

### 2.3 Isolation and analysis of axons obtained with a blue light laser

We collected axon bundles and spheroids separately for biochemical analysis after dissecting MNOs using the blue light laser. After peeling off the PDMS chip from the glass slide, axon bundles and spheroids were separately collected into tubes. For Western blotting, the collected tissues were rinsed with PBS and dissolved in a lysis buffer (TNE buffer (10 mM Tris-HCl (pH 7.5), 150 mM NaCl, 1 mM EDTA) supplemented with 5 mM sodium fluoride, 2 mM PMSF, 1 mM dithiothreitol, 1 mM sodium orthovanadate, 10 μg/mL aprotinin, 10 μg/mL pepstatin, 10 μg/mL leupeptin, 1% Triton X-100). Proteins were separated on SDS–PAGE gels and transferred to nitrocellulose membranes (Perkin Elmer, Massachusetts, United States). Subsequent Western blotting analysis was performed with a standard chemiluminescence procedure using ECL substrate (Western Lightning Plus, Perkin Elmer). Following antibodies were used: anti-TUBB3 (Beta Tubulin III, TUJ1, 1:5000, #801202, BioLegend, California, United States), anti-L1 cell adhesion molecule (L1CAM, 1:1000, #L4543, Sigma), and anti-Nucleoporin p62 (NUP62, 1:1000, #610497, BD Biosciences).

For RT-PCR, RNA was isolated with TriPure isolation reagent (#11667157001, Roche, Basel, Switzerland), and cDNA was synthesized using SuperScript IV (#18090050, Thermo Fisher). Quantitative PCR was performed with KOD SYBR qPCR Mix (#QKD-201, TOYOBO Co., Ltd., Osaka, Japan) on CFX connect (Bio-Rad Laboratories, California, United States). The following primer pairs were used: Glyceraldehyde-3-phosphate dehydrogenase (GAPDH)-F: 5′- CAT​GAG​AAG​TAT​GAC​AAC​AGC​CT-3′ and GAPDH-R: 5′-AGT​CCT​TCC​ACG​ATA​CCA​AAG​T-3’; Actin beta (ACTB)-F: 5′-ACC​ACA​CCT​TCT​ACA​ATG​AGC​T-3′ and ACTB-R: 5′-GCC​TGG​ATA​GCA​ACG​TAC​AT-3’; L1 cell adhesion molecule (L1CAM)-F: 5′-TCG​CCC​TAT​GTC​CAC​TAC​AC-3′ and L1CAM-R: 5′-ATC​CAC​AGG​GTT​CTT​CTC​TG-3’.

For the whole mount immunostaining, MNOs were fixed in 4% paraformaldehyde for 1 h, then permeabilized with 0.1% Triton X-100 in PBS for 15 min. After blocking for 1 h in PBS containing 2% normal goat serum, tissues were incubated with primary antibody over night at 4°C. Primary antibodies used were rabbit anti-TUBB3 (Beta Tubulin III, TUJ1, 1:500, mAb #5568, Cell Signaling Technologies) and mouse anti-Homeobox protein Hb9 (HB9, 1:500, sc-515769, Santa Cruz). Tissues were rinsed three times in PBS and then incubated with secondary antibodies (Alexa Fluor 488 goat anti-rabbit IgG and Alexa Fluor 647 goat anti-mouse IgG) for 2 h. After rinsing three times in PBS, nuclei were labeled with Hoechst dye for 10 min and observed using a confocal laser scanning microscope (Nikon).

## 3 Results

We use a PDMS chip on a glass slide for the generation of motor nerve organoids (MNOs; Figures 1A, B). To assess the feasibility of laser-cutting of axon bundles, we first evaluated the effects of laser-induced footprints on the culture device. First, we used a CO_2_ laser (Figure 1C), which resulted in cleavage of the whole devices. The tissues inside the culture devices were seriously damaged and not recoverable. Leakage of the culture medium was also problematic for the operation. Another attempt using a gas pulse UV laser (Micropoint, Andor Technology, Belfast, Northern Ireland) was not successful in leaving traces on the device [Note: in a separate attempt, multiple shots of the laser pulses could cleave axon bundles (data not shown), although it was time-consuming and not practical for cleaving many axon bundles.]. We then tested a blue laser cutter. In the initial attempts, the blue laser did not produce any damage or footprints on the laser-exposed sites. Interestingly, a small making with a black line on the bottom surface of a glass slide under a PDMS culture chip induced generation of burn-like footprints at the bottom of the chip (Figure 1D). During the exposure, the laser generated an orange flame-like spark at the site of exposure on the marked region, while normally the exposure site would be illuminated in blue on colorless surfaces. When we moved the laser, it continued to produce the orange spark and left a trace of a dark line on the bottom of the PDMS chip (Figure 1E). This suggests the blue laser initiates a combustion-like phenomenon at the black marked site, and the combustion continued within PDMS device and created the dark trace. We realized the followings from the attempts: i) the blue laser generally does not visibly damage PDMS, glass, or neuronal tissues; ii) the blue laser could locally damage PDMS chip if a combustion spot could be generated (e.g., with the help of black marking); iii) once the combustion spot is generated, a streak of laser-induced burn can be moved along the path of the laser.

**FIGURE 2:**
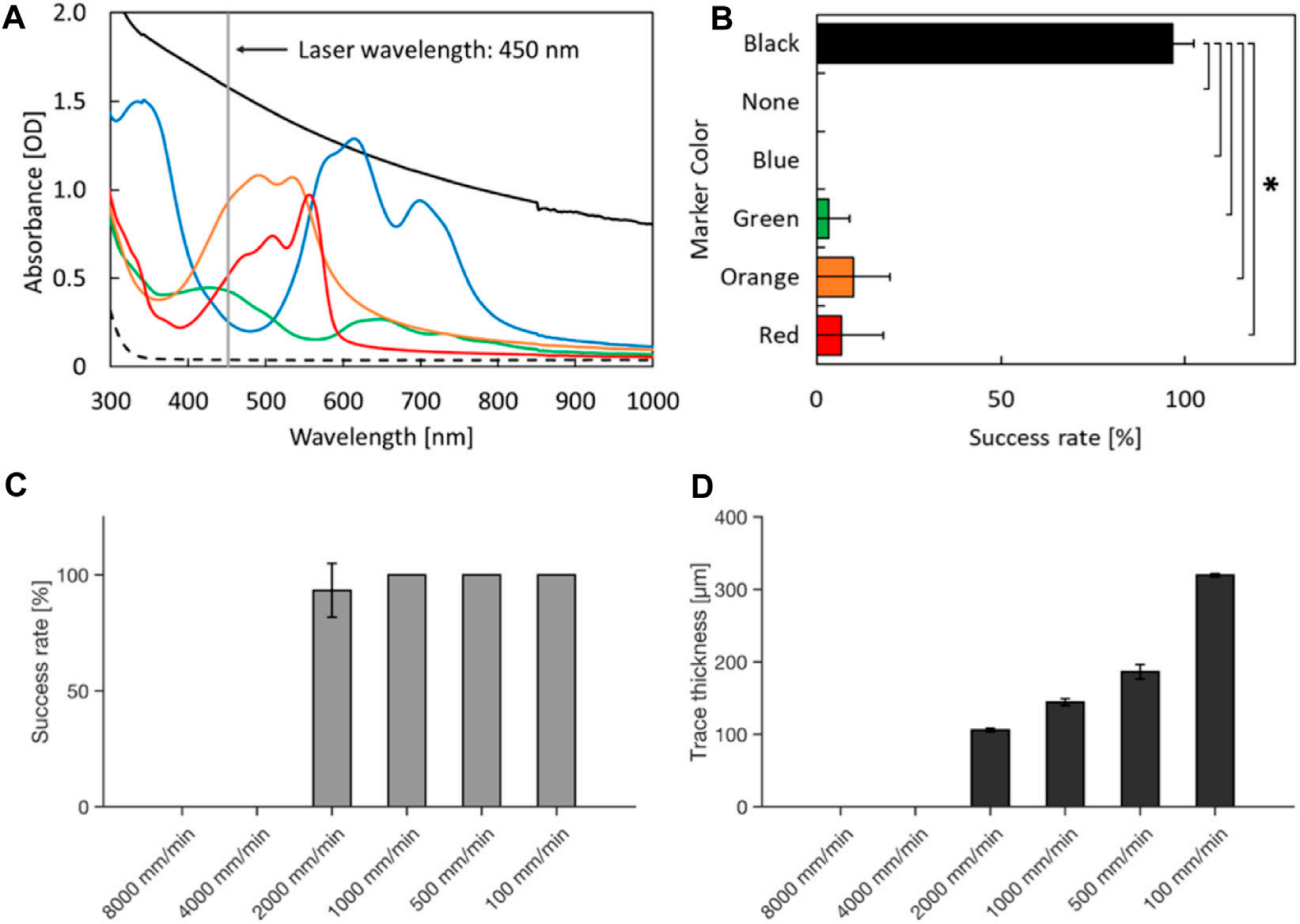
Laser-induced damage on PDMS with various color markers and cutting speeds. **(A)** Absorption spectra. The broken line indicates the absorbance spectrum of the glass on which markers were painted. **(B)** Success rate of laser-induced damage on the culture devices. *p < 0.05 (*t*-test with Bonferroni correction) and statistically significant. **(C)** Success rate of laser induced damage with different cutting speeds. **(D)** Trace thickness of laser-induced damage on the culture devices at different cutting speeds. Error bars: S.D. n = 3 for each condition.

Based on the observation, we hypothesized that the black marking converted the energy of the laser into heat, and triggered combustion-like phenomenon which can be continued along with the movement of the laser. Thus, absorbance spectrum of the marking should be important for initiation of the laser-induced burn. To investigate this, we tested whether the color of the marking alters the success rate of laser-induced damage on a PDMS chip (red, orange, green, blue, and black). According to the absorption spectra of the color markers, the black marker had the strongest absorption compared to other markers tested at 445 nm which is the wavelength of the blue laser. Orange, red, and green also showed mild absorption, but blue did not (Figure 2A). With a black marker, the combustion-like phenomenon occurred in almost all trials (Figure 2B). While orange, red, and green markers induced laser-induced burns of PDMS chip occasionally, no laser-induced damage of PDMS was observed in the experimental condition with a blue marker or no marker. These results indicate that absorbance of 445 nm light by the color markers is required for the laser-induced burn of PDMS. The local photothermal combustion phenomenon was achieved, when the laser was exposed to a thin line of black marker (about 400 μm in width), which was the thinnest line we could draw with the pen we used (Data not shown). Furthermore, the speed of cutting the PDMS chip also plays a crucial role for successful initiation of the laser-induced burn. Cutting speeds of 4,000 mm/min and above resulted in no damage induction, while speeds of 1000 mm/min and below, damage could be observed in all trials (Figure 2C). Apart from the success rate in inducing damage of the PDMS chip, the cutting speed also influences trace thickness of the damage (Figure 2D).

**FIGURE 3:**
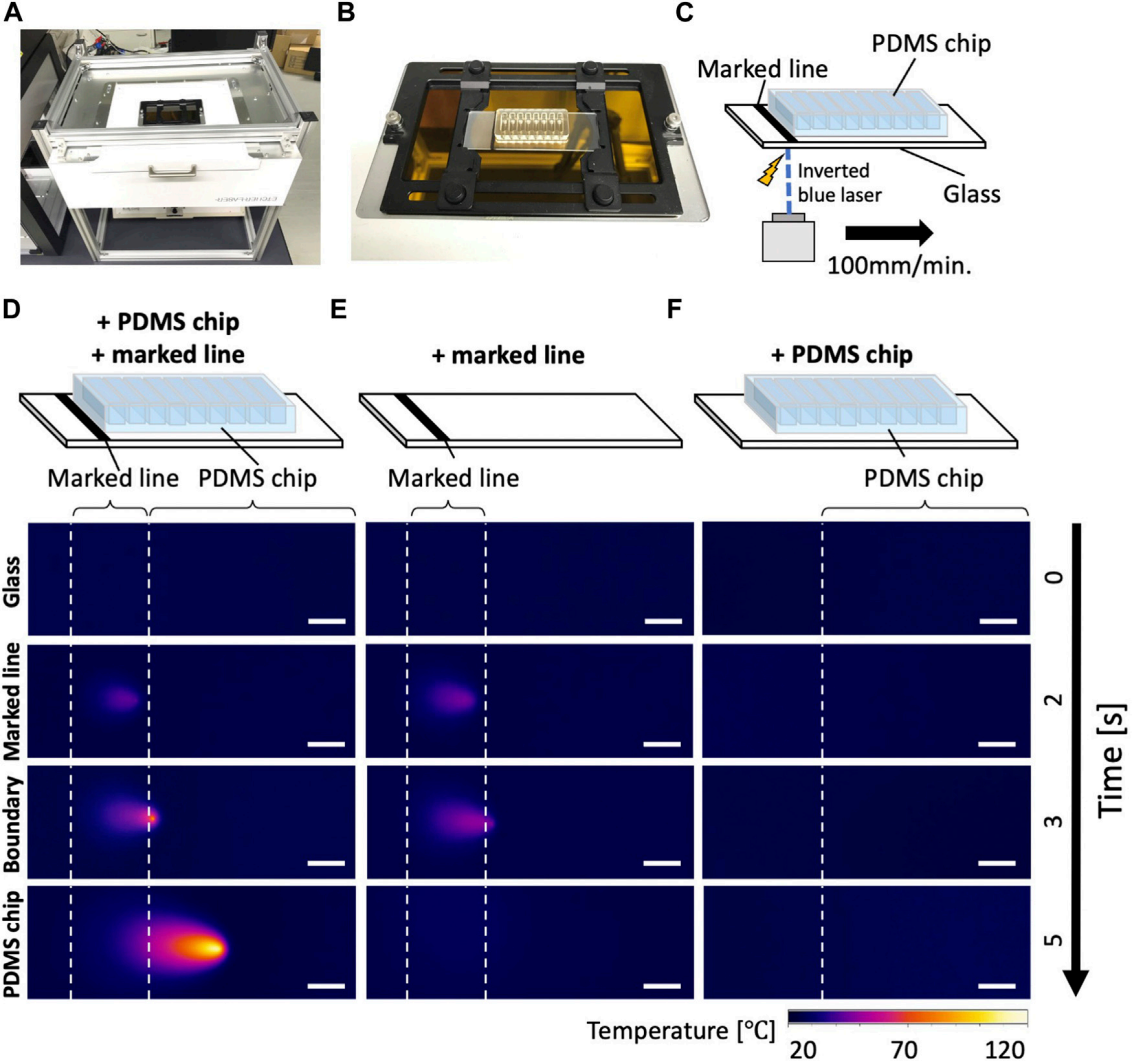
Inverted laser cutter and thermography of the laser dissection **(A)** An inverted blue laser cutter. **(B)** A sample slide holder installed on the inverted laser cutter. Note that the sample area is completely covered with a safety cover during operations to minimize laser hazard, although it is not shown in the picture. **(C)** A schematic drawing of the culture device on the laser cutter. The thermography camera (not depicted) was placed on the same side (bottom) of the glass with the laser. **(D–F)** Thermography during laser operations. Please note that the represented temperature could be underestimating the local temperature of light-induced combustion due to the heat diffusion in the glass. **(D)** A glass with a PDMS chip and marked line, **(E)** with only a marked line, **(F)** with only a PDMS chip. Scale bar: 5 mm.

To cut the PDMS chip filled with culture media from underneath, we inverted the laser cutter and installed a slide holder (Figures 3A–C). Next, we investigated how the heat produced at the black making is continued and moved along the movement of the laser. To assess the laser-induced heat, the temperature was measured with an infrared (IR) camera. On the black marker, laser increased the temperature on the exposed spot. The high temperature spot was sustained and moved along the laser movement when the PDMS chip was attached adjacent to the black marking (Figure 3D). The heat spot did not persist without the PDMS chip even if the heat spot was generated on the marker (Figure 3E). The high-temperature spot did not appear without a marker (Figure 3F). These indicate that the laser energy is converted to heat on the marker and sustained in the PDMS device. Notably, a recent study described “photothermal pyrolysis” of PDMS, which is essentially the same phenomena with our observations described above (Shin et al., 2021).

**FIGURE 4:**
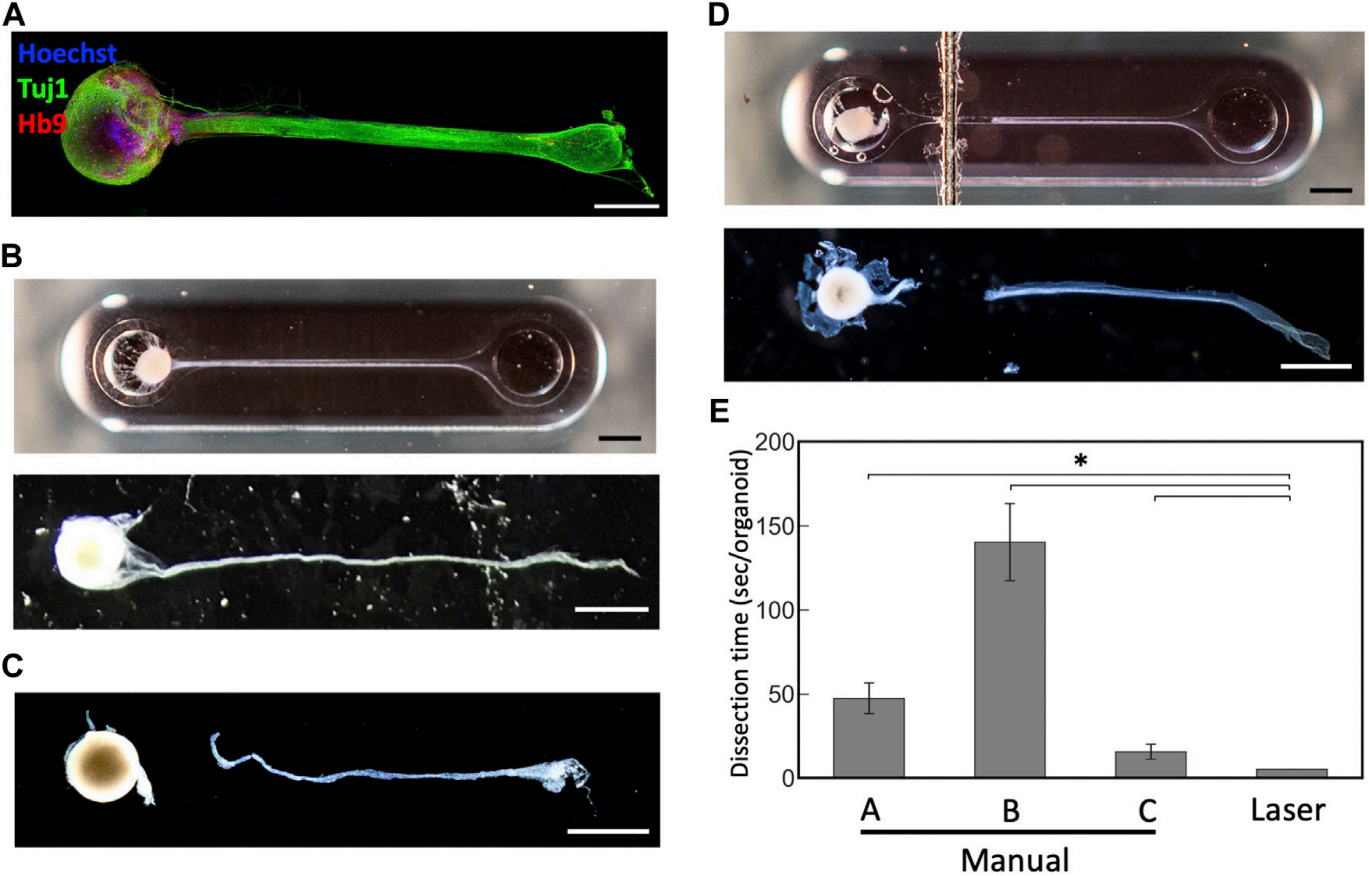
Dissection of motor nerve organoids with a blue laser **(A)** Immunostaining of motor nerve organoid after 48 days in culture. **(B)** An intact motor nerve organoid before and after removal from device. **(C)** A motor nerve organoid after manual dissection. **(D)** A motor nerve organoid after laser dissection before and after removal from device. **(E)** Dissection time per organoid. Organoids were dissected manually by three experimenters **(A–C)** and with the blue laser. Scale bars: 1 mm. Error bars: S.D. n = 6 for each condition. *p < 0.05 (Paired *t*-test with Bonferroni correction; manual cutting V.S. laser cutting) and statistically significant.

To test whether this technique can be applied to cut axon bundles of MNOs generated in the PDMS device, we cultured MNOs and subjected for laser exposure. They were cultured for at least 4 weeks in the device and formed thick axon bundles. They expressed neuronal marker beta 3 tubulin (Tuj1; TUBB3) and motor neuron marker HB9 (Figure 4A). We did not observe Hoechst dye-stained nuclei in the distal (>1 mm from spheroid) segment of axon bundles. Once the bundles were formed (Figure 4B), tissues were subjected to the laser cutting. We applied black marking adjacent to the tissues in the PDMS chip. We irradiated the blue laser to the marking, then moved the laser to the tissues. We aimed the laser at the proximal (<1 mm from spheroid) segment of the axon bundles. The laser made a clean cut of the axon bundles from the spheroids, similar to axons cut manually (Figures 4C, D). Axon bundles were separated out from spheroids without obvious damage to the axon bundles or spheroids, and no burns or shrinking were observed. Importantly, the dissection using the blue laser was significantly faster compared to manual dissection (Figure 4E). The laser-induced cleavage of axon bundles leaves a dark trace on the PDMS chip at the exposure site (Figure 4D), but it did not significantly damage the PDMS chip and can hold the culture media within the device, resulting in an easy operation after the laser-induced cleavage. Axons were not damaged by the blue laser when no black marking near the tissues were applied (Data not shown). After the laser-induced cleavage, the axon bundles and spheroids were collected for further analysis, as they could be differently affected by the manual and laser cleavage operations.

**FIGURE 5:**
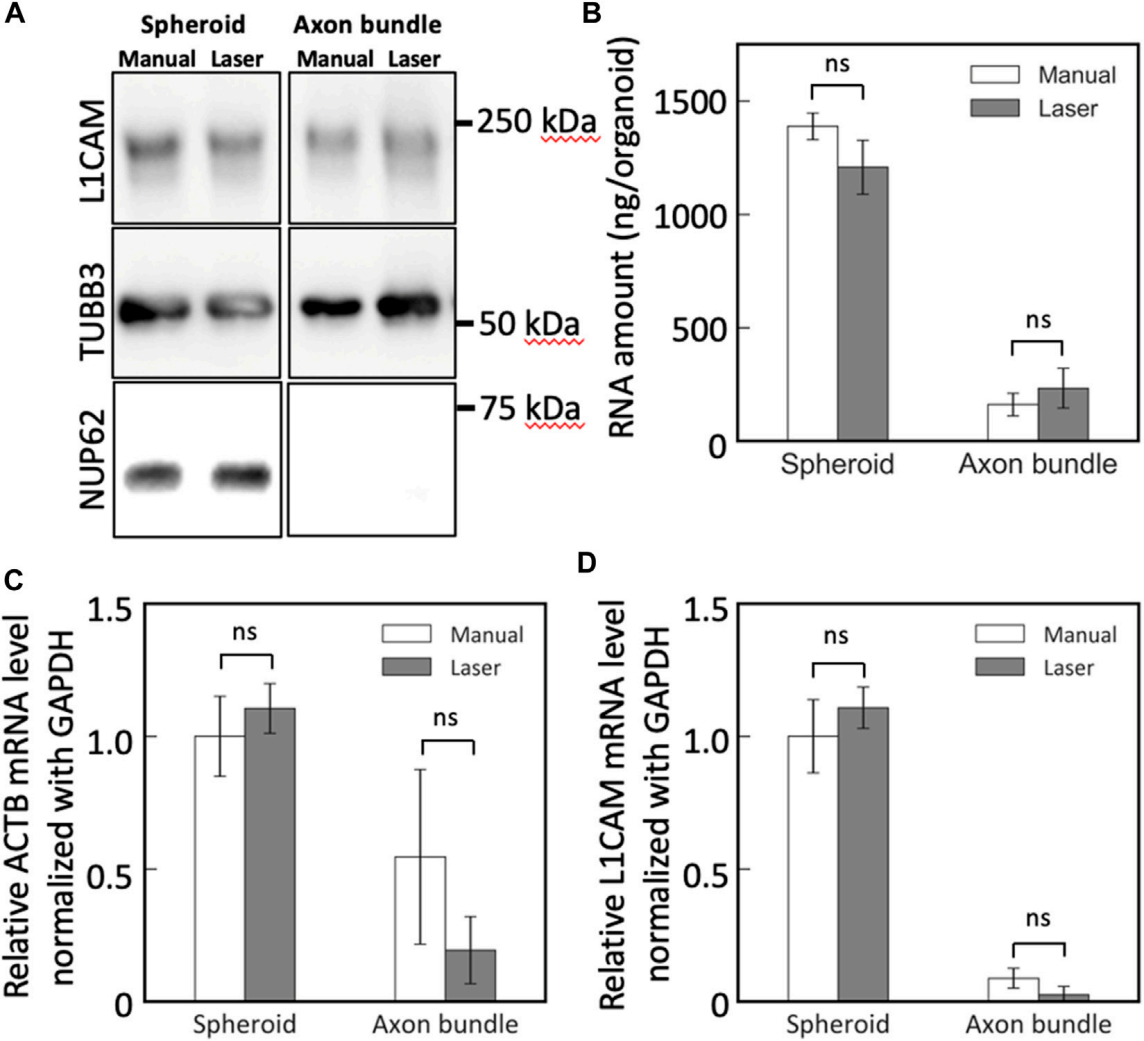
Western blotting and RT-PCR analysis of spheroids and axon bundles isolated from motor nerve organoids. **(A)** Protein lysates were analyzed with antibodies against L1CAM, TUBB3, and NUP62. **(B)** Collected total RNA amount from spheroids and axons. **(C,D)** Relative mRNA abundance of **(C)** ACTB and **(D)** L1CAM. Error bars: S.D. n = 3 for each condition. ns: *p* > 0.05 (Paired *t*-test with Bonferroni correction) and not significant statistically.

The collected neural tissues from MNOs were lysed and subjected to downstream biochemical assessments. Bradford assay revealed that the lysates of the axon bundles and spheroids dissected with the blue laser contained similar amount of protein in lysates obtained by manual dissection of axon bundles and spheroids from MNOs in average (manual dissection: spheroid 0.4 mg, axon bundle 0.03 mg, laser dissection: spheroid 0.44 mg, axon bundle 0.02 mg per organoid, respectively). With Western blotting, neuronal beta 3 tubulin TUBB3 and L1CAM could be found in both axon bundles and spheroids, but nuclear protein NUP62 was not detected in axon bundle-derived protein lysates (Figure 5A). No significant difference was observed in the amount and quality of the detected proteins, indicating that the laser cutting does not induce biochemical damage of axon bundles and compatible for biochemical assays.

Next, we obtained RNA from the spheroids and the axon bundles after cutting the MNOs manually or with the blue laser. The dissection methods did not make significant difference on the RNA yield for both spheroids and axon bundles (Figure 5B). With RT-PCR, we quantified the relative abundance of mRNAs in spheroids and axon bundles dissected with hands or a laser (Figures 5C, D; Table 1). The relative amount of mRNAs was comparable in manually cut and laser-cut axon bundles and spheroids, indicating that the laser dissection method does not degrade RNAs in the axons. RT-PCR Ct values were comparable between manual and laser-dissected axon bundles and spheroids for all three tested genes. Taken all this together, the dissection with a blue light laser has the capability to isolate the axon bundles and spheroids from MNO without disturbing biochemical molecules in axons.

**TABLE 1:** Ct value of RT-PCR.

Gene	Manual spheroid	Laser spheroid (ns)	Manual axon bundle	Laser axon bundle (ns)
GAPDH	19.3 ± 0.2	19.1 ± 0.3	24.0 ± 0.7	25.5 ± 1.2
ACTB	18.5 ± 0.1	18.3 ± 0.4	24.3 ± 1.5	27.4 ± 2.2
L1CAM	23.9 ± 0.2	23.6 ± 0.4	32.2 ± 1.2	34.4 ± 0.2

Data are expressed as mean. ± S.D., The comparison of Ct value was performed using paired t-tests; manual cutting V.S., laser cutting. ns: *p* > 0.05.

## 4 Discussion

Here, we present a method for rapid harvesting of axon bundles from nerve organoids. The radiation of blue visible light laser on the black marker enables easy dissection of neural tissues in the culture device through a combustion-like phenomenon.

Using this method, axon bundles can be rapidly isolated with less effort than manual dissection without hindering the yield or quality for biochemical analyses. While other methods such as microfluidic devices using microgrooves (Nijssen et al., 2019) reported RNA amounts per device comparable to that of a single cell, nerve organoids can give much larger amounts (approx. 200 ng per organoid) of axonal RNA. The RNA and proteins obtained by the laser dissection was not impaired in quality as far as we could examine with RT-PCR and Western blotting, respectively. By using the method presented, we can gain axons quickly and consistently, which is important for the outcome of biochemical assays in general. The speed could contribute to scaling up the assay, as processing time is critical for quality of the biological specimen for downstream analyses.

The cost of the blue laser system used in the study was around 1000 USD, which is normally used for cutting and engraving papers, woods, leathers, etc. The laser unit includes a XY positioning system (scanning stage) and a control software. The CO_2_ laser compared to the blue laser was around 3000 USD, which also includes a positioning system. They are much more affordable than a UV laser that we tested (about 40,000 USD, data not shown), and professional laser systems do not include XY stages. We flipped the blue laser upside down to cut the axons in liquid, which cost extra of about 500 USD, which is still marginal to costs of professional systems. Therefore, this method is resource-efficient since it only requires a laser cutting machine and markers.

We applied black ink to our culture device with a dry-erase pen. We drew lines to apply the ink, since it was easy to control. However, the shape of the black markings could be altered, as the local combustion could be initiated, as long as the blue laser is exposed on the markings before exposed to a PDMS chip. The shape of the markings could be altered depending on the application.

The use of this technique may not be limited to nerve models but could also be useful to cut other multicellular tissues. In this study, we focused our attention on obtaining axons, however, more physiological axon bundle tissues could be obtained by co-culturing the tissue with other cell types including glial cells. In the future, it would be even more beneficial to use the laser dissection on the axon bundles cocultured with other cells to facilitate biochemical and cellular analysis of more physiological models of axons and nerves *in vitro*. The method could potentially be used for other thin biological tissues cultured within a PDMS chip.

Limitations of the methods include relatively low resolution of the laser dissection. Faster speed (2000 mm/min) of the laser operation could reduce the width of the laser-induced combustion to around 100 microns, but it hindered the success rate slightly. It would be necessary to make the heat induced spot or streak smaller to cut finer structures, although the method is suitable for cutting the axon bundles. We did not examine the structure of the cut surface of the axons processed with the presented method. Since we did not observe any defects of proteins or RNAs, we believe that little damage is caused on the biological molecules in axons. It would be interesting how well the axons are quickly “sealed” at the dissected ends, which could be beneficial to capture biological molecules within the axoplasm. We have not tested if the method could be applied to larger structures (e.g., neural organoids and spheroids). It will expand the usage of the method if it could be applied to a bigger structure.

## 5 Conclusion

The radiation of blue visible light laser on the black marker enables easy dissection of neural tissues in the culture device through a combustion-like phenomenon. Using this method, axon bundles can be rapidly isolated with less effort than manual dissection without hindering the yield or quality for biochemical analyses. This simple method provides an efficient alternative to manual dissection for biochemical assessment of axons.

## Data Availability

The raw data supporting the conclusion of this article will be made available by the authors, without undue reservation.
